# Measuring Safety: The Lifetime Sense of Safety Scale in Kenyan Adults

**DOI:** 10.1002/alz70857_104733

**Published:** 2025-12-25

**Authors:** Anne Nyambura Njogu, Anne Njoki Gitere, Levi A. Muyela, Catherine Bikeri Onyancha, Lucy Wambui Kamau, Kendi Muchungi, Catherine Ajalo, Litha Musili, Jasmit Shah, Rachel W Maina, Zul Merali, Chinedu Udeh‐Momoh, Karen Blackmon

**Affiliations:** ^1^ Brain and Mind Institute, Aga Khan University, Nairobi, Kenya; ^2^ Aga Khan University, Nairobi, Nairobi, Kenya; ^3^ Wake Forest University, School of Medicine, Winston‐Salem, NC, USA; ^4^ Dartmouth Hitchcock Medical Center, Lebanon, NH, USA

## Abstract

**Background:**

Safety is central to brain health; yet, there are currently no tools to assess perceived safety. Here, we describe preliminary psychometric data from a novel Lifetime Sense of Safety (LSS) scale in healthy Kenyan adults. The LSS evaluates self‐perception of safety across childhood and adulthood settings, cumulatively referred to as “the protectome.

**Method:**

The 6‐item LSS scale was collaboratively developed in English and Kiswahili. Respondents are asked to retrospectively evaluate perceived safety in childhood at (1) home, (2) school, and (3) neighbourhood, as well as in adulthood at (4) home, (5) work, and (6) neighborhood, using a color bar with a corresponding number range from “0” (lowest) to “10” (highest) sense of safety. The LSS scale was administered to 163 Kenyan adults (97 f; 66 m), ranging in age from 35 to 80 years (m=53; sd=10), with broad educational attainment (primary to doctoral level). We evaluated internal consistency, factor structure, sex effects, and convergent validity between LSS total score and measures of perceived stress (PSS‐4) and resiliency (Connor Davidson Resiliency Scale ‐ CDRS).

**Result:**

Data were normally distributed and internal consistency was good (α = 0.86). Suitability for factor analysis was established by sampling adequacy (KMO=0.81) and item cross‐correlations (Bartlett's test=455; *p* < 0.001). Exploratory factor analysis with oblimin rotation revealed two latent factors. Childhood items 1‐3 exhibited high loadings on Factor 1 (0.83‐0.95), while adulthood items 4‐6 displayed high loadings on factor 2 (0.72‐0.91). Results support the LSS scale as a measure of two latent factors (childhood and adulthood safety) accounting for 75% of total variance. There were no sex effects on latent factors. The LLS total score was negatively correlated with PSS total score (*r* = ‐0.30; *p* < 0.001) and positively correlated with CDRS total score (*r* = 0.26; *p* <0.001).

**Conclusion:**

The LSS scale is a psychometrically sound measure of “perceived safety”, with 2 latent factors reflecting childhood and adulthood safety. Perceived safety is negatively associated with stress and positively correlated with resiliency, which supports use of the LSS scale in investigations of the lifetime “protectome” on late‐life brain health.

